# Genome-Wide Maps of Circulating miRNA Biomarkers for Ulcerative Colitis

**DOI:** 10.1371/journal.pone.0031241

**Published:** 2012-02-16

**Authors:** Radha Duttagupta, Sharon DiRienzo, Rong Jiang, Jessica Bowers, Jeremy Gollub, Jessica Kao, Keith Kearney, David Rudolph, Noor B. Dawany, Michael K. Showe, Tom Stamato, Robert C. Getts, Keith W. Jones

**Affiliations:** 1 Applied Reasearch Group, Affymetrix Inc, Santa Clara, California, United States of America; 2 Research and Development, Genisphere LLC, Hatfield, Pennsylvania, United States of America; 3 Lankenau Institute for Medical Research, Wynnewood, Pennsylvania, United States of America; 4 Division of Gastroenterology, Lankenau Medical Center, Wynnewood, Pennsylvania, United States of America; 5 The Wistar Institute, Philadelphia, Pennsylvania, United States of America; I2MC INSERM UMR U1048, France

## Abstract

Inflammatory Bowel Disease – comprised of Crohn's Disease and Ulcerative Colitis (UC) - is a complex, multi-factorial inflammatory disorder of the gastrointestinal tract. In this study we have explored the utility of naturally occurring circulating miRNAs as potential blood-based biomarkers for non-invasive prediction of UC incidences. Whole genome maps of circulating miRNAs in micro-vesicles, Peripheral Blood Mononuclear Cells and platelets have been constructed from a cohort of 20 UC patients and 20 normal individuals. Through Significance Analysis of Microarrays, a signature of 31 differentially expressed platelet-derived miRNAs has been identified and biomarker performance estimated through a non-probabilistic binary linear classification using Support Vector Machines. Through this approach, classifier measurements reveal a predictive score of 92.8% accuracy, 96.2% specificity and 89.5% sensitivity in distinguishing UC patients from normal individuals. Additionally, the platelet-derived biomarker signature can be validated at 88% accuracy through qPCR assays, and a majority of the miRNAs in this panel can be demonstrated to sub-stratify into 4 highly correlated intensity based clusters. Analysis of predicted targets of these biomarkers reveal an enrichment of pathways associated with cytoskeleton assembly, transport, membrane permeability and regulation of transcription factors engaged in a variety of regulatory cascades that are consistent with a cell-mediated immune response model of intestinal inflammation. Interestingly, comparison of the miRNA biomarker panel and genetic loci implicated in IBD through genome-wide association studies identifies a physical linkage between hsa-miR-941 and a UC susceptibility loci located on Chr 20. Taken together, analysis of these expression maps outlines a promising catalog of novel platelet-derived miRNA biomarkers of clinical utility and provides insight into the potential biological function of these candidates in disease pathogenesis.

## Introduction

Inflammatory Bowel Disease (IBD) represents - a chronic relapsing disorder of the gastrointestinal (GI) tract affecting over 6.6 million individuals in the US and Europe [1]. A significant risk factor for colorectal cancer, IBD is stratifiable into two disorders: Crohn's Disease (CD) and Ulcerative Colitis (UC) [2]. Both these subtypes are characterized by inflammation of the digestive tract, with CD involving widespread inflammation of all layers of the GI tract while UC is characterized by localized inflammation of the colon. Current modalities for the diagnosis of both the subtypes involve a combination of invasive endoscopic procedures and diverse clinical indices that provide only relative measures of disease severity and outcome. In the absence of well-established diagnostic gold standards for IBD, there is a collective interest in the identification of novel clinical biomarkers that are cost-effective, afford rapid turnover and provide needed insight into disease complexity and biology [3], [4].

The accessibility of nucleic acids in circulation and their emerging value in correlating disease burden to clinical outcomes provides an important paradigm for disease surveillance and therapeutic management [5]. Described first in 1948 in human blood [6], extra-cellular nucleic acids exist abundantly as both DNA and RNA species. Despite enigmatic biological functions, global characterization of their distributions, size and genomic origins have established them as discriminating indicators of a range of tumor associated genomic, epigenetic and transcriptional change [5], [7]. Within the various classes of cell-free circulating nucleic acids, a significant fraction of the genomic content is composed of miRNAs [8]. Representing a highly stable and conserved class of ∼22 nt long endogenous non-coding transcripts, miRNAs represent approximately 1–2% known genes in eukaryotes [9] and function to negatively regulate gene expression through base-pairing to target mRNAs [10]. Currently over 1000 mature miRNAs are annotated in the human genome [11], [12] with the potential to post-transcriptionally regulate about 30% of all protein coding genes [13]. This interleaved regulatory organization encompasses a vast array of cellular and developmental cascades with an increasing number of miRNAs being correlated to deregulation of these processes [14]. The oncogenic and tumor suppressive functions of miRNAs are now well-established with mechanisms ranging from copy number changes, epigenetic silencing and modification of transcriptional control of miRNA loci [15]. Specific examples encompass cancers of both hematopoietic and non-hematopoietic origin – thus making this species a promising and tractable candidate for diagnostic screening [7], [8]. Biologically, circulating miRNAs survive the extracellular catalytic environment through sequestration within sub-cellular particles such as micro-vesicles and exosomes [7]. Current models for their functionality involve trafficking through circulation to facilitate long–range communication between different inter-cellular sites.

A key component of the biology of inflammatory bowel disease (IBD) involves a deregulated immune response against intra-luminal bacterial antigens leading to intestinal tissue injury. In recent years, a collection of expression profiling studies have provided compelling evidence for the role of miRNAs in modulating crucial steps of the immune response [16]. Specific examples include varied roles of miR-16, miR-17∼92 and the miR-146 family in modulating different aspects of innate and adaptive immunity. Additionally, evidence from auto-immune disorders such as rheumatoid arthritis, psoriasis and systemic lupus erythematosus further endorse the diversity of miRNA functions in various immune cascades [16]. Although the specific role of miRNAs in intestinal diseases are poorly understood, loss of intestinal miRNAs in mouse models are known to result in impaired epithelial barrier function resulting in acute inflammation [17]. Taken together, this evidence suggests that the fundamental physiological role of miRNAs in inflammatory disorders, such as IBD, is to maintain normal intestinal homeostasis.

Given that the pathology of IBD involves a complex interplay of immune and non-immune cells, it is of interest to understand the impact of different classes of blood-derived miRNAs as indicators of disease physiology [18]. Importantly, platelets, representing an abundant class of circulating non-immune cells classically engaged in blood hemostasis are now increasingly recognized as critical drivers of cell-mediated enteric immune response that lead to the infiltration of inflammatory cells into the intestinal lumen [18], [19], [20]. As a first step towards understanding the contribution of circulating miRNAs of specific lineages in IBD, we have explored through microarray analysis, miRNA expression levels in micro-vesicle, PBMC and platelet fractions from a cohort of 20 normal and 20 affected individuals diagnosed with Ulcerative Colitis. Using a Significance Analysis of Microarrays approach [21], [22] we can identify profiles of differentially expressed miRNAs in each fraction. Furthermore, through a non-probabilistic binary linear classification using Support Vector Machines (SVM) [23], we can segment the dataset to delineate a 31 marker platelet-derived miRNA signature that has 92.8% accuracy, 96.2% specificity and 89.5% sensitivity in stratifying UC patients from normal individuals. This panel can be validated successfully through qPCR assays. Additionally, correlation analysis of intensity levels across the patient-control strata reveal subgroups of tightly clustered miRNAs – suggestive of potential functional relationships through coordinate regulation of miRNA expression. Taken together, this data suggests that platelet-derived biomarkers are compelling clinical predictors of disease outcome; consistent with the emerging role of platelets as critical players in the pathology of UC. Examination of biological pathways targeted by these miRNAs reveal enrichment of genes involved in modulation of cellular architecture and regulatory cascades implicated in cell-mediated immune response. These global expression maps thus provide a necessary and critical resource for biomarker development and begin to provide needed perspective into the extensive regulatory networks influencing the clinical expression of this disease.

## Results

### Biomarker Derivation from circulating miRNA profiles in different hematological fractions

**Figure 1:**
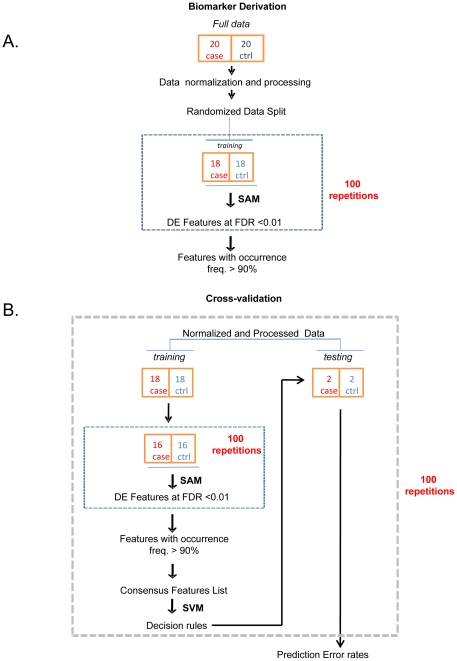
Workflow for biomarker derivation and computation of predictive estimates. (A) Differentially expressed miRNAs were first derived by SAM and those exhibiting a FDR of <1% and frequency of occurrence >90% in 100 iterations, were selected as biomarkers. (B) The decision rules were obtained by SVM classification and the predictive estimates of the selected biomarkers determined by 10-fold cross-validation.

**Figure 2:**
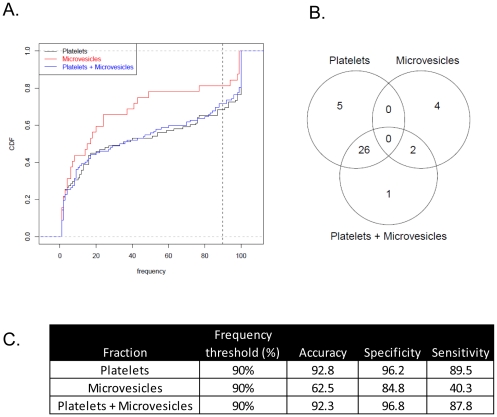
Characteristics and performance measures of biomarkers derived from Platelets, Micro-vesicles or Platelet/Micro-vesicle combined fraction. (A) Cumulative distribution frequency (CDF) plots of biomarkers derived from the different fractions. The dotted line represents the 90% cut-off frequency across the study population (B) Counts and overlaps of miRNA biomarkers derived from the platelet, micro-vesicular and combined fraction (C) Performance measures of biomarkers from each fraction selected at the 90% frequency threshold.

Given that miRNAs have been shown to reliably classify disease from normal population and are important mediators in the IBD mediated inflammatory response, we chose to investigate the changes in blood-derived miRNA spectrum in a cohort of Ulcerative Colitis patients. Blood samples from 20 patients and 20 normal individuals, characterized in Table 1 were collected with informed consent. The case versus control cohorts did not significantly differ by ethnicity, age, gender or familial cancer incidences. A statistically significant disposition to Gastrointestinal (GI) disorders (i.e., Gastritis, IBD, Crohn's Disease, Colitis) was found in the patient cohort (8 versus 0 individuals, P = 0.0016 by one-sided Fisher's Exact Test) while the control patients demonstrated a greater incidence of non-GI disorders such as diabetes, coronary diseases, thyroid disorders, hypertension (6 versus 0 individuals, P = 0.01 by one-sided Fisher's Exact Test) (Table 1). To enable identification of inflammation-mediated changes the samples were specifically sub-fractionated into PBMC (Peripheral Blood Mononuclear Cells) and platelets. The purity of the isolated fractions were verified by quantitative PCR amplification of either the leukocytic mRNA CD45 or platelet specific gene product Glycoprotein IIB [24] and show a statistically significant and clear separation of the two fractions (data not shown). Additionally, micro-vesicular sub-fractions were isolated to identify markers that were detected free in circulation. Total mRNA from all three sub-fractions (PBMC, micro-vesicles and platelets) were extracted and independently profiled on the Affymetrix miRNA arrays containing 847 human miRNAs. For derivation of miRNA biomarkers that were differentially expressed (DE) each individual hematopoietic fraction was initially analyzed independently. For each analysis, the datasets were split 90∶10 with 18 individuals randomly selected from each arm of the study and control cohorts (Fig. 1A). Quantile normalized and processed data from all three fractions (Fig S1) were analyzed using Significance Analysis of Microarrays (SAM) and Differentially Expressed (DE) features selected at a False Discovery Rate (FDR) threshold of 1% (Fig. 1A and Fig S2). A total of 6, 11 and 52 miRNAs were identified as differentially expressed from a single iteration of SAM across the PBMC, micro-vesicular and platelet fractions, respectively (Table S1). We find that the majority of these signatures to be dominated by profiles of up-regulated miRNAs, with 92% and 100% of miRNAs demonstrating increased expression in the platelet and micro-vesicular fractions, respectively. Interestingly, we find all miRNAs derived from the PBMC isolates to display down-regulated expression suggesting distinct biology of each of these isolated miRNA populations. In order to select miRNAs useful for stratifying patients from controls, the derivation procedure was repeated 100 times with random re-sampling of the data to minimize the dependency on a single dataset. Finally, the occurrence of the DE features obtained from this iterative process was counted and features demonstrating a frequency no less than 90% selected as potential biomarkers (Fig. 1A and Fig. 2A). A signature of 31 platelet-derived, 6 microvesicle-derived and 0 PBMC miRNAs were obtained through this process (Fig. 2B and Tables S2 and S3). In order to determine if a combination of the fractions led to a more discriminative signature the entire derivation process was re-run using a union of platelet and micro-vesicle fractions. PBMC were excluded since there were no differentially expressed miRNAs within that fraction alone. We isolated a list of 29 biomarkers from this concatenated group and mapped the overlap between the different lists (Fig. 2B and Fig S2D). We find that 89.6% (26/29) miRNAs obtained from the combined fraction (derived from platelet and micro-vesicle) overlap with biomarkers derived from the platelet fraction only (Fig. 2B, Tables S2 and S4). A subset of only 6 miRNAs are found to be unique between these two datasets with hsa-miR-423-3p exclusive to the platelet-micro-vesicle combined fraction and hsa-let-7i-star, hsa-miR-1271, hsa-miR-1296, hsa-miR-31 and hsa-miR-345 identified from the platelet derived fraction only. Additionally, we can also co-identify 33% (2/6) miRNAs (hsa-miR-202 and hsa-miR-1263) between the independent micro-vesicle and the micro-vesicle/platelet combined fractions. The remaining set of 4 miRNAs representing 66% (4/6) of the dataset and comprised of hsa-miR-628-5p, hsa-miR-603, hsa-miR-221-star and has-miR-455-3p are unique only to the group of micro-vesicular miRNAs derived from independent analysis of this fraction (Fig. 2B, Tables S3 and S4). Taken together, this data characterizes two major signatures of differentially expressed miRNA biomarkers of hematopoetic origins in Ulcerative Colitis – those that are principally derived from the platelet fraction and a minority subset of micro-vesicular ancestry.

**Table 1:** Demographics of individuals recruited for this study.

		Control	Patient
**Total**		20	20
**Ethnicity**	Caucasian	15/20 (75%)	13/20 (65%)
	Others	5/20 (25%)	4/20 (20%)
	Unknown	none	3/20 (15%)
**Gender**	F	13/20 (65%)	14/20 (70%)
	M	7/20 (35%)	6/20 (30%)
**Age (yrs)**		42.7±12.9	49.5±11.9
**Average age at diagnosis (yrs)**		N/A	35.5±11.9
**Family Medical History**	Cancer	3/20 (15%)	4/20 (20%)
	others (non GI)	6/20 (30%)	none
	others (GI)	none	8/20 (40%)
**Medical History**	Cancer	2/20 (10%)	none
	Ulcerative Colitis	none	20/20 (100%)
	CD	none	none

Gender is designated as either M (Male) or F (Female). Disorders are designated as GI (Gastro-intestinal) or CD (Crohn's Disease).

### Development and validation of recurrent miRNA predictors

To estimate the predictive capability of these signatures, each of the biomarker categories were subjected to non-probabilistic binary linear classification using Support Vector Machines (SVM) (Fig. 1B). Measurement of classifier success was assessed through a 10-fold cross-validation method. A cohort of randomly selected 18 case-control subjects were chosen as the training set while 2 individuals each from the two enrollment categories were reserved for testing. The training set was further stratified to randomly sub-select 16 case-control individuals that were subject to the same feature selection process using SAM, originally used in biomarker derivation (Fig. 1B). The prediction error rates were then estimated based on application of the SVM classifier to the test set (Fig. 2C and Fig S3). Through an iterative cycle of 100 repetitions the performance measures (confusion matrix of true positives, true negatives, false positives and false negatives) were computed and recorded for the three principal classes (platelets, micro-vesicle and their union). The aggregated summary statistics for these 3 fractions demonstrate that the best classifier performance is obtained from the 31 miRNAs derived from the platelet fractions that can classify patient from control individuals with 92.8% accuracy, 96.2% specificity and 89.5% sensitivity (Fig. 2C). The platelet and micro-vesicle derived class comprising of 29 miRNA biomarkers similarly significantly predicted disease outcome with 92.3% accuracy, 96.8% specificity and 87.8% sensitivity (Fig. 2C). The performance measures of micro-vesicle biomarkers in contrast had the least predictive powers (62.5% accuracy, 84.8% specificity and 40.3% sensitivity) (Fig. 2C). None of these performance estimates were significantly correlated to independent biometric variables such as age, gender, height or weight though regression analysis (data not shown). Misclassification estimates of the training data by the final classifier reveal that only 4 diseased individuals in the micro-vesicle derived category (p14, p15, p18, p21) were anomalously categorized as normal. In contrast these estimates were much more conservative in the platelet-derived or platelet-micro-vesicle combined classes with respectively only 1 (p3) and 0 individuals, being inaccurately classified, respectively (Fig S4). These categorization and performance measures thus indicate that the principal statistical power in classification of disease versus normal individuals is driven by the platelet-derived miRNA biomarker class. This category of biomarkers therefore represents the most discriminating candidates with the best diagnostic potential.

### Independent validation of levels of specific circulating miRNA biomarkers

**Figure 3:**
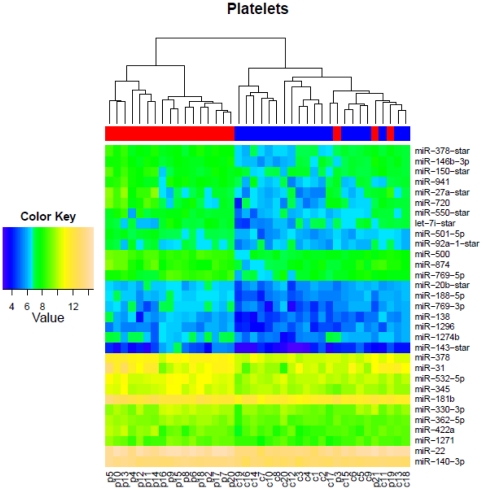
Comparison of expression levels of the platelet-derived miRNA biomarkers in the patient and control cohorts. Unsupervised hierarchical clustering of samples (controls in blue: C1–C20 and patients in red: P2–P21) based on summarized intensity values from the 31 differentially expressed miRNA biomarkers. The log_2_ intensity values are shown in the Color Key bar scale.

**Figure 4:**
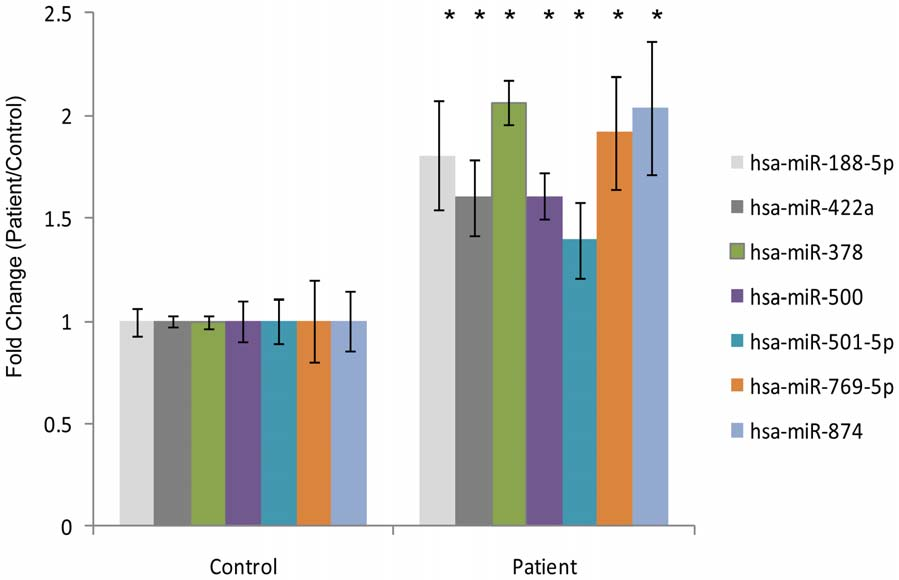
Validation of expression levels of miRNA biomarkers derived from the platelet fraction. miRNA expression levels of 7 biomarkers (n = 4, *P values<0.001) were validated by qPCR. The p values are calculated based on a Student's t-test of the replicate 2∧ (−ΔCt) values for each miRNA in the control group (normal individuals) and test groups (patients).

We next sought to gain an improved understanding of the biological levels and validity of the platelet-derived biomarker panel derived from classifier analysis. The differentially expressed profiles of the 31 platelet-derived miRNAs (Fig. 3 and Fig S5A) demonstrating the highest predictive performance was analyzed through unsupervised hierarchical clustering. The expression map of the 31 candidate miRNAs displayed a clear separation of the patient versus control groups (Fig. 3) with 3 patients (p3, p19 and p21) being misclassified by the clustering algorithm. The relatively higher proportion of misclassified individuals obtained through this analysis compared to classifier estimates obtained by SVM (3 vs 1 individual) indicate that unsupervised hierarchical clustering does not separate the samples as well as the SVM algorithm. The majority of miRNAs in this panel displayed an average log_2_ signal intensity magnitude of >6 with only 23% (7/31) target miRNAs demonstrating lower abundance processed log_2_ signal intensity <6 (Fig S5A). Similarly, the major proportion of biomarkers derived from the platelet and micro-vesicular combined fraction (72% or 21/29 miRNAs) display comparable log_2_ intensity distributions of 6 or greater. Both the micro-vesicle specific miRNAs in this biomarker panel are low abundant with an average log_2_ signal intensity of 4.8 for hsa-miR-202 and log_2_ < = 6 for hsa-miR-1263 (Fig S5B). These differences are visualized through unsupervised hierarchical clustering of the data which also identify misclassification of the same 3 individuals (p3, p19 and p21) as was observed in the group of platelet-derived miRNAs (Fig S6). In order to independently validate specific candidate biomarkers, we selected sub categories of miRNAs from the platelet-derived biomarker panel for verification by quanititative PCR (qPCR) assays. First, 24 of the 31 marker panel were identified based on 100% representation in the cross-validation iterations. These were divided into 4 quartiles based on p-values, mean intensity and fold changes. A union of all of these categories were taken and eight representative candidate biomarkers (hsa-miR-188-5p, hsa-miR-22, hsa-miR-422a, hsa-miR-378, hsa-miR-500, hsa-miR-501-5p, hsa-miR-769-5p, hsa-miR-874), with distributions ranging from marginally below the mean to the maximum intensity measurable on the array (log_2_ intensity values ranging from 7.6 to 13.6 for this frequency measure) were randomly chosen for validation in pooled platelet samples derived from patient and control cohorts (Fig. 4). Our results demonstrate that 88% (7/8) of all the differentially expressed candidates validated successfully and demonstrated a fold change difference ranging from 1.4–2.04 on comparing patients over controls (Fig. 4). These estimates match the array estimates with no significant difference between the two platforms (p-value of 0.6 from paired Student's t-test). Taken together this data indicates the capability of the selected biomarker panel to clearly delineate patient and control samples with a high degree of confidence.

### Segregation of biomarker panel into miRNA clusters

**Figure 5:**
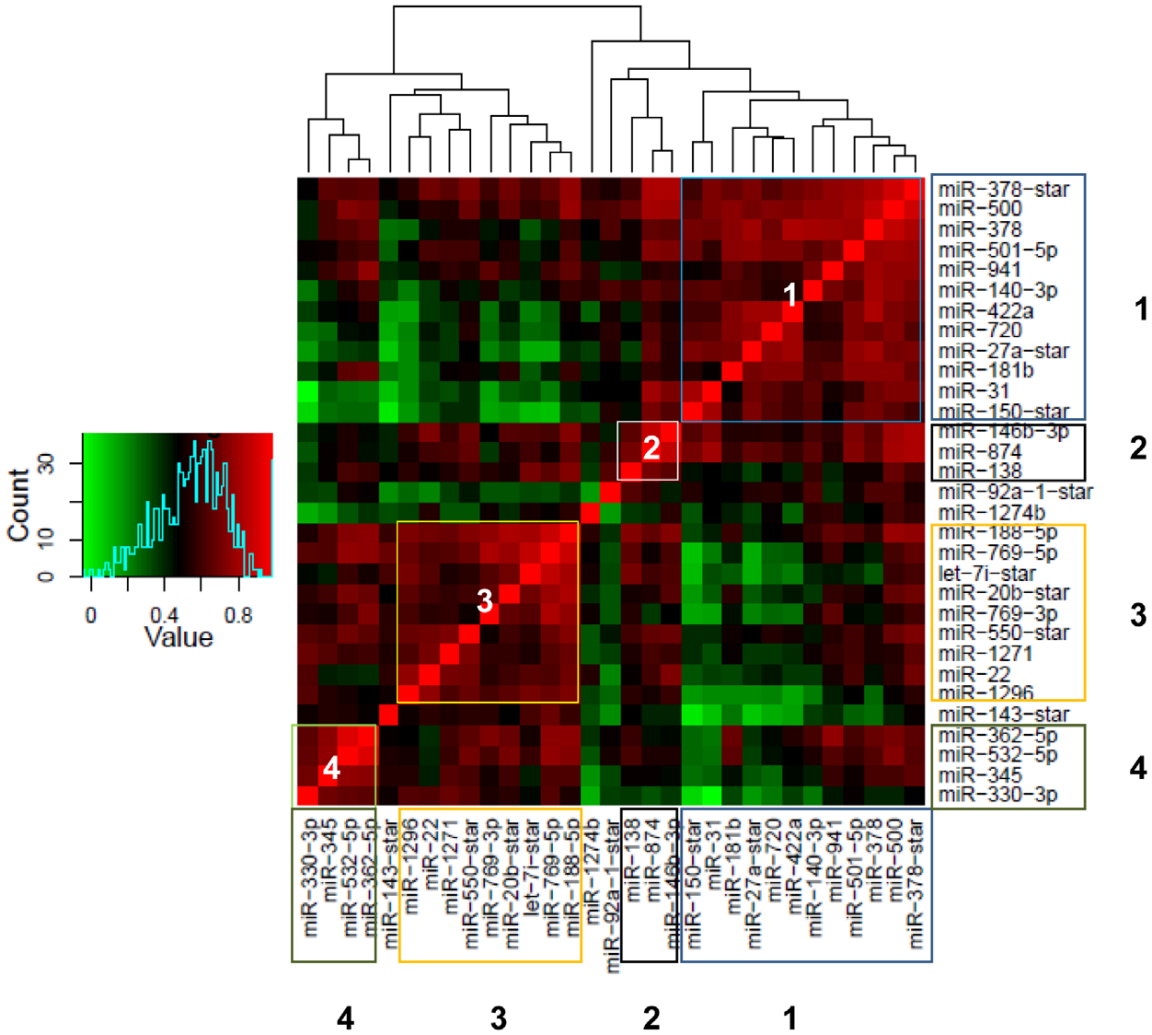
Sub-clusters of platelet-derived miRNA biomarkers. Unsupervised hierarchical clustering of miRNA biomarkers based on the Pearson correlation coefficients among the miRNA expression profiles from 20 patients and 20 controls. The coefficient values are shown in the bar scale. Each of the 4 main clusters with significant correlation values are indicated in boxes.

In order to examine possible functional relationships amongst the miRNA members of the platelet biomarker panel, we next analyzed correlation of expression patterns between the 31 selected species through comparison of log_2_ intensities across the patient and control strata. Our results revealed a clear separation of 90% of the data or 28/31 of the biomarkers into 4 highly correlated clusters with an average Pearson correlation coefficient of 0.75 and p-value<3.95E-05 (Fig. 5 and Table S5). For one of the largest clusters (Cluster 1) (Fig. 5 and Table S5) we can also additionally identify miRNA candidates that co-cluster based on genomic location (i.e. hsa-miR-500 and has-miR-501-5p that reside closely on Chr. X and hsa-miR-27a-star and hsa-miR-150-star originating from Chr. 19). Co-localization is observed for two additional candidate miRNAs in Cluster 3 that are processed products of the same pre-miRNA: hsa-mir-769-5p and hsa-mir-769-3p; both localized on Chr. 19. Positional concordance is not observed for any of the remaining markers (Table S5). This analysis therefore identifies subsets of miRNAs that demonstrate correlated expression across the samples, with additional chromosome-dependent physical association seen for some selected pairs.

### Identification of gene pathways associated with miRNA biomarker signatures

To understand the biological relevance of the diagnostic signatures we next wanted to determine the spectra and response of potential mRNAs targets of the identified platelet-derived biomarkers. To address this, predicted targets of the 31 miRNAs were first computationally identified through Target Scan 5.0 [13], [25], [26] and DIANA microT v3.0. No targets could be identified for the biomarker hsa-miR-92a-star– and hence this was excluded from the analysis. A total of 5493 conserved targets were identified through this process (Table S6) and a subset of 3304 non-redundant mRNA targets of 30/31 biomarkers were selected and subjected to Gene Ontology (GO) or Ariadne Ontology and pathway classification using Pathway Studio software. Biologically relevant groups were identified by analyzing for significant shared ontology terms. Our analysis revealed that the biomarker targets were significantly enriched in genes associated with transcriptional categories such as transcription factor activity, regulation of transcription nucleotide binding and actin-based cytoskeleton assembly (p-value<1.33×10^−8^) implying a biological role of these miRNAs in the regulation of these processes (Table 2 and Table S7). Significantly, we find approximately 27% of the regulated targets (526 genes) to be involved in the regulation of transcription, 32% (65 genes) participating in actin-based cytoskeleton assembly and between 9–14% (15–54) of genes engaged in diverse lipid metabolism pathways (Table 2 and Table S7). Additionally, through the analysis of annotated signaling pathways we also find enrichment of signaling pathways that participate in both cytoskeleton regulation and immune-mediated inflammatory response. Specifically we observe between 24–58% overlap of miRNA targets with signalling cascades such as TNF-α or T-cell activation pathways known to be involved in triggering the innate immune response (Table 2 and Table S7). Taken together this data indicates that genic targets of diagnostic miRNA biomarkers of Ulcerative Colitis are selectively enriched in candidate genes that engage in aspects of the pro-inflammatory response observed in the pathophysiology of this disease.

**Table 2:** Gene ontology (GO) classification and pathway analysis of predicted mRNA targets of the 31 platelet-derived biomarkers.

Annotation	Term	Genome annotation	Overlap^#^	% Overlap	p-value
Actin-based cytoskeleton assembly	Ariadne Ontology	202	65	32	2.74E-10
Transcription factors		955	203	21	1.33E-08
Vesicular secretory pathway		93	33	35	5.69E-07
TGFBR		16	9	56	0.000143
Regulation of transcription	Biological Process	1937	526	27	2.41E-125
Transcription		2282	517	22	5.18E-90
Nucleotide binding	Molecular Function	2265	462	20	1.50E-43
Transcription factor activity		1215	297	24	1.53E-42
DNA binding		2430	476	19	4.89E-40
Kinase activity		783	207	26	1.09E-34
Transferase activity		1547	325	21	1.40E-32
Actin binding		375	86	22	2.18E-11
Actin Cytoskeleton Regulation	Signaling Pathways	548	176	32	3.10E-16
NTRK→AP-1/CREB/ELK-SRF/MYC/SMAD3/TP53		95	45	47	7.75E-11
Gonadotrope Cell Activation		722	197	27	2.24E-10
FGFR→AP-1/CREB/CREBBP/ELK-SRF/MYC		98	44	44	1.09E-09
EGFR→AP-1/CREB/ELK-SRF/MYC		93	42	45	2.11E-09
ThrombopoietinR→AP-1/CREB/ELK-SRF/MYC		85	39	45	4.57E-09
ThrombinR→AP-1/CREB/ELK-SRF/SP1		110	46	41	7.58E-09
VEGFR→AP-1/CREB/MYC		87	39	44	1.03E-08
FibronectinR→AP-1/ELK-SRF/SREBF		104	44	42	1.05E-08
EphrinR→actin		216	74	34	1.12E-08
PDGFR→AP-1/MYC		77	35	45	3.83E-08
EGFR/ERBB3→MEF/MYOD/NFATC/MYOG		130	50	38	4.63E-08
Focal Adhesion Regulation		322	98	30	5.07E-08
EGFR→AP-1/ATF2		82	36	43	7.40E-08
TNFRSF1A→AP-1/ATF/TP53		36	21	58	1.01E-07
T Cell Activation		951	233	24	1.78E-07
EGFR/ERBB2→TP53		81	35	43	1.82E-07
IGF1R→MEF/MYOD/MYOG		62	29	46	2.65E-07
VasopressinR2→MEF/MYOD/NFATC/MYOG		123	46	37	4.20E-07
ErythropoietinR→AP-1/CREB/MYC		80	34	42	4.39E-07
VasopressinR2→CREB/ELK-SRF/AP-1/EGR		128	47	36	5.88E-07
Glycerophospholipids and ether lipids metabolism	Metabolic Pathways	372	54	14	2.65E-09
Sphingolipid metabolism		206	25	12	0.001593
Metabolism of triacylglycerols		103	15	14	0.002504
Glycosylphosphatidylinositol-anchor biosynthesis		136	18	13	0.002855
Pterine biosynthesis		227	22	9	0.038294

Overlap^#^ refers to the number of gene annotations intersecting with genome annotations. The top ranking GO terms, % overlaps and the counts of overlapping entities ranked by the most significant p-values are listed.

## Discussion

Diagnostic assessment and therapeutic management of IBD spectrum disorders currently derives from a combination of clinical examination, laboratory indices and invasive procedures [27]. Given the emerging association of circulating miRNA species with a spectrum of physiological and disease conditions [7] we have systematically explored the utility of secretory miRNAs isolated from different hematological fractions as non-invasive predictors of Ulcerative Colitis.

To address this question, we have investigated broadly across distinct whole blood fractions comprising both cellular (platelets, PBMC) and acellular compartments (exosomal/micro-vesicular) that are known to express and shuttle miRNA cargo through circulation. A set of distinct, differentially expressed maps of annotated miRNAs have been constructed from a cohort of 20 diseased versus 20 normal individuals through hybridization to miRNA arrays. Fraction-specific differentially expressed miRNA signatures have been interrogated through SAM [21] at a 1% FDR [22] that identify primarily up-regulated miRNA candidates in the full dataset with only a minority of miRNAs displaying down-regulation of expression. Through molecular classifiers derived from a Support Vector Machine model and a multiple random sub-sampling strategy, our results (Fig. 2C) have isolated a distinct diagnostic signature of 31 miRNAs with increased expression that derive specifically from the platelet fraction and can stratify UC patients from normal individuals with 92.8% accuracy, 96.2% specificity and 89.5% sensitivity. Given the low number of differentially expressed miRNAs from the PBMC and micro-vesicular fractions, no predictive miRNA species could be identified from the PBMC isolates and only a poorly performing minority 6-marker panel derived from independent analysis of the micro-vesicular fraction. Additionally, no added benefit is obtained from combination of the platelet and micro-vesicular strata which generate a 29 marker panel consisting 27 platelet-derived and 2 micro-vesicular markers with comparative predictive performance in distinguishing case from control (92.3% accuracy, 96.8% specificity and 87.8% sensitivity). Given the significant overlap observed between the platelet-derived and platelet+micro-vesicle-derived profiles (Fig. 2B) and the poor performance metrics of the non-platelet miRNAs we conclude that the origin of the majority of disease-specific predictors in UC map back to the anucleate platelet fraction. Additionally, since classifier analysis did not identify any down- regulated miRNA biomarkers, this data also suggests that the primary physiological response in Ulcerative Colitis is to enhance expression of miRNA subsets. The divergence of platelet vs. micro-vesicular signatures is interesting and indicates distinct sub-cellular addresses for these miRNA species. Taken together, this supports the hypotheses that the isolated micro-vesicle fraction mapped in this study either do not originate from platelet progenitors or very selectively sort platelet derived miRNA cargo into classes destined for trafficking through the hematopoietic circuit. Given the significant overlaps observed in previous studies between platelet and micro-vesicular signatures [28], this disparity may be indicative of disease specific characteristics of Ulcerative Colitis. The validity of the identified platelet-derived class can be additionally verified with 88% success through qPCR assays run on pooled patient and control samples (Fig. 4) – thus providing independent confirmation of the differential regulation of this panel. Furthermore, these markers segregate into 4 highly correlated intensity clusters, often containing specific pairs of miRNAs that share proximity of sub-chromosomal location (Table S5). Cumulatively these results suggest distinct sets of biomarkers that may be coordinately regulated or share functional regulatory sub-networks closely synchronized under the given physiological conditions.

The biology of IBD spectrum disorder is known to encompass a complex interplay of innate and adaptive immunity that ultimately regulates a regional intestinal inflammation response [29]. Circulating blood platelets representing non-immune cells and engaged classically in the hemostatic function of surveying vessel integrity are emergent mediators of active inflammation modulating local intestinal tissue injury [19], [20]. Enriched in a small but diverse transcriptome [30], platelets contain a large proportion of mature miRNA that can potentially engage in the regulation of gene expression. In order to understand the diversity of our identified miRNA biomarker panel, we have assessed the overlap between our maps and miRNA profiles from normal or disease-specific signatures surveyed across different studies. These analyses identify between 6% (2/31) to 29% (9/31) overlap between the 31-platelet-derived biomarker panel with miRNAs derived from platelet populations of normal individuals [24], [28], [31] and no overlap with UC specific miRNAs profiled from peripheral blood [32]. Additionally comparison to miRNA maps of non-platelet origin involved in a broad spectrum of inflammatory disorders [16] reveal only two overlapping miRNAs: hsa-miR-140 and hsa-miR-146b with the majority of the 31 marker panel thus being exclusive to our study. Taken together, these comparative analyses clearly delineate that members of the platelet-derived 31 biomarkers are (a) enriched in miRNA species that are typically not detected in the normal platelet transcriptome, (b) are highly compartment specific and detectable only on enrichment of the platelet fraction and (c) display specificity of expression restricted to Ulcerative Colitis compared to other inflammatory disorders.

Given that miRNAs are known to be collective regulators of approximately 30% of human genes [13] understanding of the mechanism of gene regulation mediated through the 31-miRNA biomarker panel is of importance. The prevalence of biological functional processes was evaluated through analysis of GO terms on 3304 non-redundant predicted mRNA targets representing approximately 13% of the estimated number of protein coding genes in the human genome [33]. Stratification of the top canonical groups and pathways reveal enrichment of genes engaged in actin-cytoskeleton assembly, vesicular secretary pathway, biosynthesis and metabolism of diverse lipids such as glycerol phospholipds, pterin and tri-acylglycerol, implicated in a variety of cell-regulatory and immune stimulation roles (Table 2). Specific examples include (a) regulation of the members of the Rho-ROCK pathway involved in cytoskeletal reorganization/cell adhesion response to wound healing ([34] and references therein), (b) involvement of sphingolipids as modulators of growth factor receptors and second messengers of known agonists of the inflammatory response such as tumor necrosis factor-α, interleukin-1β [35], (c) regulation of Pterin metabolism- in response to IFN-γ stimulation [36], (d) genes involved in ionic transport and regulation of calcium homeostasis that may influence membrane permeability and (e) modulators of classic inflammatory regulators such as TNF-α. The predicted mRNA target spectrum is also dominated by transcription factors/mediators such as PI3K, KLF5, etc. that we hypothesize play a role in modulation of key genes involved in the regulation of the other diverse functions of metabolism, biosynthesis, transport, etc. [37]. Additionally, we also find that a subset of the biomarkers (i.e., hsa-miR-1271, hsa-miR-874, hsa-let-7i-star and hsa-miR-20b-star) can target ATGs16L, a gene necessary for autophagy and implicated in IBD susceptibility [38], [39]. Therefore, comparison of these targets to published studies [34], [37], [40], [41] reveal a high degree of concordance in functional groups but often limited overlaps in terms of specific gene targets – suggesting that the landscape of physiologically relevant mRNAs in IBD is far from being saturated. Therefore, this study complements our current understanding of the UC transcriptome and provides interesting insight into the expression divergence of this disorder.

In principal, the platelet derived miRNAs could contribute to the pathophysiology of UC through regulation of the platelet transcriptome, consequently influencing downstream interaction with the intestinal mucosa. Adhesion of platelets to the mucosal endothelium is known to occur though a CD40/CD40L contact-dependent process resulting in platelet activation [19], [20] and a chemokine mediated immune response. The ultimate outcome of this trigger is the recruitment of leucocytes to the endothelium which results in a focal inflammatory response. These mechanisms are consistent with the functional classes of mRNA target genes identified in this study and implicated in the modulation of cellular architecture and inflammation. Taken together, these results support a hypothesis which offers a direct role for miRNAs in influencing platelet activation and cell-cell mediated immune response in gut inflammation.

Given that micro-vesicles are known mediators of cell-cell communication [42] it is intriguing to speculate if the miRNA content of platelets can potentially be transferred to endothelial cells through vesicular fusion. In this model, the epithelial transcriptome would be directly targeted by the identified platelet miRNA regulators. Analysis of differentially expressed miRNA profiles from colonic epithelial tissues from UC patients can isolate 34% (30 out of 89) significantly down-regulated mRNAs [37] corresponding to representative targets of 57% (17 out of 30) miRNAs from our biomarker panel. Comparison of fold change densities of these anti-correlated target mRNAs against all down-regulated transcripts on the array, reveal a statistically significant difference (p-value of 0.011 by Binomial test and 0.007 by Fisher Exact Test) between the two groups (Fig S7). This suggests that the platelet miRNAs could possibly be a significant factor in the down-regulation of endothelial gene expression in the context of IBD.

Given the prevalence and increasing disease-specific role of miRNAs, it is of interest to catalogue the association of these sequences with naturally occurring polymorphisms in polygenic disorders [43]. Mechanistically, single nucleotide changes can have significant functional impact through either impeding miRNA biogenesis, or through inhibition of binding to target miRNA sequences. Examples of such functional polymorphisms are numerous [44] (http://www.patrocles.org/Patrocles_miRNAs.htm) and has been demonstrated to impact a variety of disease states [45], [46], [47], [48]. We can map polymorphisms in the mature miRNA sequence of 35% of the miRNAs (11 of 31) in our biomarker signature and 20% (673 out of 3304) of their computationally predicted mRNA targets (http://compbio.uthsc.edu/miRSNP/). It is intriguing to speculate whether the patient who is misclassified by the platelet-derived miRNA signature is impacted by this type of variation. A link between polymorphisms and miRNAs were also explored by examining possible associations of the 31-biomarker prognostic panel with IBD susceptibility loci identified through genome-wide association studies. We can identify a close physical link (220 Kb) between hsa-miR-941 and rs2297441, a SNP mapping to the 20q telomere that is associated with high confidence to UC [49]. As more evidence demonstrating the significance of polymorphisms affecting miRNA synthesis, binding, stability or functional activity accumulates, it will become important to investigate these changes in the context of disease associations and diagnostic accuracy of miRNA biomarkers.

The abundance and stability of miRNAs in circulation holds the promise of easy and rapidly accessible biomarkers. Given the prognostic and diagnostic utility of this class in cancer and the inherently vast regulatory potential of miRNA molecules, we have assessed the ability of fraction-specific miRNAs in delineating incidences of Ulcerative Colitis. To our knowledge the current results outline for the first time platelet-specific miRNA signatures for an inflammatory disorder, with exceedingly high predictive measures. The potential of these biomarkers to influence gene expression in context of modulating intestinal homeostasis is explored and the spectrum of putative physiological targets evaluated for their regulatory potential. In summary, results obtained from this study support a model where expression and coordinate control of various polygenic components of UC pathogenesis may be mediated by the interplay of circulating miRNA species and their physiological targets. As universally applicable tests are developed a combinatorial use of these indicators would perhaps provide the most rational strategy for disease determination and clinical therapy.

## Materials and Methods

### Ethics statement

The study cohort consisted of 20 anonymized patients recruited from the office of Main Line Gastroenterology. Additionally, 20 normal samples were obtained from the volunteer staff of the Lankanau Institute of Medical Research and Lankenau Hospital. Written informed consent was obtained from all patients prior to obtaining samples and medical history. The Main Line Hospital's Institutional Review Board approved of this study.

### Fractionation of whole blood into Micro-vesicle, PBMC and Platelet fractions and isolation of RNA

Whole blood (7 to 9 ml) was collected from patient and control individuals in BECTON-DICKINSON 16×100 mm 10.0 mL BD Vacutainer® plastic EDTA blood collection Tubes (Becton & Dickinson, Franklin Lakes, NJ). To protect against RNA degradation samples were treated with Baker's yeast RNA (Sigma R6750) within 5 minutes of draw to give a final concentration of 1.25 mg/ml and then diluted with equal volume of PBS. Samples were separated into the different hematopoietic fractions by density gradient centrifugation. Briefly, the diluted blood samples were layered over Ficoll-Paque™ plus (GE Healthcare) at a 3∶4 ratio by volume and centrifuged at 400 g for 30 to 40 minutes at 25°C. This process resulted in a fractionation of the sample into plasma (upper layer), PBMCs and platelets (narrow central band), and a band of erythrocytes and granulocytes at the bottom of the tube. The upper plasma layer was removed, centrifuged at 100,000 g for 1 hr at 4°C using a Beckman Ti50 rotor and the pellet isolated to generate the **micro-vesicular fraction**. This fraction consists of microparticles, exosomes, and other cellular debris. To fractionate the PBMC from Platelets, the Ficoll-Paque layer containing these cellular populations was removed, and 3 volumes of 1× PBS added to the layer and the mixture centrifuged at 65 g for 15 min at room temperature. The supernatant fraction was saved and the pellet re-suspended in 10 ml PBS and re-centrifuged under the same conditions. The resulting pellet was isolated to represent the high density **PBMC population**. The supernatant fractions from the first and second washes were pooled and centrifuged at 450 g for 20 min at room temperature to isolate the pellet representing the **platelet fraction**. All three pellet fractions were mixed with 1 ml of Trizol-LS reagent and total RNA extracted according to the Trizol procedure.

### Labeling and hybridization of platelet, micro-vesicle and PBMC samples to the Affymetrix miRNA Arrays

Total RNA ranging in concentration from 1 ug–3 ug was labeled using the Genisphere HSR labeling kit (P/N HSR30FTA) and hybridized overnight to the Affymetrix Genechip miRNA array (P/N 901326). The arrays were washed and stained using standard Affymetrix protocols and scanned using the Affymetrix GCS 3000 7G Scanner. Feature intensities were extracted using the miRNA_1-0_2xgain library files.

### Quantitative RT-PCR

Assays to quantify differential expression of miRNAs in patients vs. healthy controls were performed using the ABI 7300 real-time PCR instrument and miScript quantitative PCR System (Qiagen). Pooled Patient (P2–P21) and Control (C1–C18 and C20) samples were used in duplicate Reverse Transcription (RT) reactions. Approximately 10 ng of cDNA was used in duplicate PCR assays for each RT. The normalizers used for this analysis were: Hs_RNU6B_3, Hs_miR-320b_2, Hs_miR-543_1and Hs_miR-654-3p_2. For analysis of the purity of the platelet fraction, patient and control pools from each of the plasma-derived classes of platelets and PBMCs were similarly tested using 66.6 ng of total RNA and QuantiFast™ SYBR® Green RT-PCR system (Qiagen). The platelet specific gene ITGA2B (Glycoprotein IIB) and the leukocyte specific CD45-common antigen marker PTPRC were used as target mRNAs and 18S (RRN18S) and Actin (ACTB) used as normalizers. All oligonucleotide sets were purchased from Qiagen.

### Datasets

The datasets described in this manuscript are MIAME compliant and have been deposited in NCBI's Gene Expression Omnibus Database (http://www.ncbi.nlm.nih.gov/geo/).

The data series is accessible through GEO Series accession number GSE32273:http://www.ncbi.nlm.nih.gov/geo/query/acc.cgi?acc=GSE32273.

### Analysis of the miRNA data

**Data Preprocessing:** Data preprocessing was performed via Affymetrix miRNA QC Tool, which consisted of extraction of raw intensities for each individual feature followed by background subtraction based on GC content of anti-genomic probes, transformation of values through addition of a small constant (value 16), quantile normalization and finally median summarization of all probe sets for each feature. The intensity data used in all analysis are log_2_ transformed. Triplicates available for one case (p7) and one control (c9) for each of the fractions under study were analyzed by utilizing the mean intensity values.

**Software Packages:** All statistical analysis was performed under the R programming language environment (www.r-project.org). The R “samr” package was used for Significance Analysis for Microarray (SAM) and the R “e1071” package was used for Support Vector Machine (SVM) with the non-linear radial basis function as the kernel. The unsupervised Hierarchical clustering algorithm was implemented in the R “hclust” function and generated using Euclidean distance matrix and complete-linkage agglomeration. All heat maps were generated using the R “gplots” package.

**miRNA Biomarker Selection:** Biomarker selection was based on 100 iterative cycles of SAM run on re-sampling of the full 20 patient and 20 control sample set through a randomized 9∶1 data split (i.e., a ‘training set’ of 18 cases and 18 control individuals per iteration). Each individual fraction (platelet, micro-vesicle or PBMCs), or the combination of all three fractions were separately analyzed. Differentially expressed (DE) miRNA features were identified at a False Discovery Rate of 1% and features occurring at a frequency greater than 90% (consensus features) selected as potential biomarkers.

**Cross-Validation to Estimate Prediction Error Rates:** Support Vector Machines (SVM) was used for classifying the IBD cases from controls. To assess the prediction error rates a 10-fold cross-validation procedure was applied (Fig. 1). Briefly, the full data set of 20 cases/controls was randomly sampled to generate a training set of 18 cases/controls and a testing set of 2 cases/controls. The training set was further split into a random subset of 16 cases/controls and the biomarker derivation process run iteratively through SAM to select the consensus features followed by SVM classification. The decision function obtained by SVM classification was applied to the testing set and the confusion matrix (e.g., counts of false positives and false negatives) recorded. The entire procedure was repeated 100 times and the prediction error rates estimated from the aggregated confusion matrix.

**Inter-feature correlation analysis among the 31 Biomarkers:** The correlation structure among the 31 platelet-derived biomarkers was examined by calculating the Pearson's correlation coefficients among the 31 miRNAs based on their normalized expression profiles across all cases and control individuals. Clusters were defined as subgroups exhibiting minimal correlation coefficients >0.6 and were identified by visualizing the heat map plotted by unsupervised hierarchical clustering.

**Prediction of miRNA targets and enrichment analysis:** For prediction of mRNA targets, the 31 platelet derived biomarkers were first separated into two categories based on the star designation. Targets were determined using either DIANA-microT 3.0 algorithm for the star miRNA sequences (http://diana.cslab.ece.ntua.gr/) or TargetScan Human v5.0 (http://www.targetscan.org/cgi-bin/targetscan/data_download.cgi?db=vert_50) for the non-star miRNA sequences. Targets were selected based on conservation of families/sites from Target Scan and a precision score greater than 0.4 from DIANA. A total of 5493 mRNA targets were identified out of which 3304 unique mRNAs were selected after removing redundancies and subjected to pathway exploration using Pathway Studio software from Ariadne Genomics (http://www.ariadnegenomics.com/). Using this software and its accompanying Gene Ontology and Interaction databases, incidence of predicted miRNA targets were matched against the target collection and the top-ranking pathways and Gene Ontology groups selected. The statistical enrichment for each of these pathways/groups was computed by the similarity score or p-value calculated as a ratio of a number of common objects between two pathways to the total number of objects in them.

**Correlation Analysis of miRNA and mRNA Expressions:** To identify expression changes in putative target tissue mRNAs, genome–wide mRNA expression data from colonic pinch biopsies of Ulcerative Colitis patients were analyzed [37]. The pre-processed raw intensities for 12,258 probes (excluding the control probes from the array) were extracted from patient-control cohorts (4 normal individuals and 5 affected Ulcerative Colitis patients). Differentially expressed mRNAs were identified by SAM at an FDR <0.1% and genes exhibiting a fold change >2 selected for further analysis. The numbers of differentially expressed genes were counted for both the whole array and the miRNA targets and enrichment of down-regulated target mRNAs assessed by either the Binomial test or the Fisher's Exact test. The null hypothesis for this analysis tests that the proportion of down-regulated miRNA target genes is equivalent to the fraction of down-regulated genes predicted not to be miRNA targets. The alternative hypothesis tests that the proportion of down-regulated miRNA target genes is larger than the non-target miRNA genes.

## Supporting Information

Figure S1 **Box plot of log_2_ signal intensity distribution of human miRNAs (white) and background probes (red) for 20 patients and 20 controls for each fraction (A) Platelets (B) Micro-vesicles (C) PBMC after background subtraction, quantile normalization and median summarization.** The black bar represents the median of each distribution and the dashed lines represent the box–plot range set by the whiskers.
(TIF)

Figure S2 **Analysis of differentially expressed miRNA biomarkers from (A) Platelets (B) PBMC (C) Micro-vesicular and (D) Combined Platelet and Micro-vesicle fractions.** Comparison of observed vs. the expected scores obtained by performing Significance analysis of microarrays on all 847 miRNAs from 20 patients and 20 controls. Each miRNA is represented by a point (open circle), and the differentially expressed miRNAs represented as red (for up-regulated) or green (for down-regulated) points in the graph. The dashed line represents a FDR threshold of 1%.
(TIF)

Figure S3 **Performance estimates for the different fractions isolated in this study at a frequency cutoff of 90%.** The Combined fraction denoted by asterisk is representative of the union of all the 3 different fractions of PBMC, micro-vesicles and platelets. The different error rate calculations are given by FPR (False Positive Rate), FNR (False Negative Rate), PPV (Positive Prediction Value) and NPV (Negative Prediction Value).
(TIF)

Figure S4 **Table of predicted and observed values from each analyzed fraction based on Confusion Matrix derived from SVM on the full data comprising 20 cases and 20 controls.**
(TIF)

Figure S5 **miRNA expression levels in patient and control cohorts.** (A) Box plot of processed log_2_ intensity distributions of 31 miRNA biomarkers from Platelet fraction or (B) 29 miRNAs from the Platelet/Micro-vesicle combined fraction. The black bar represents the median of each distribution and the dashed lines the box-plot ranges. The open circles represent the outliers. The patient samples are denoted by red (cases) and normal samples by blue (controls). The platelet-derived miRNAs are denoted with the prefix ‘pl’ and the micro-vesicular miRNAs are designated as ‘mv’.
(TIF)

Figure S6 **Comparison of expression levels of miRNAs biomarkers in the patient and control cohorts derived from the combination of platelet and microvescicle fractions.** Unsupervised hierarchical clustering of samples (controls in blue: C1–C20 and patients in red: P2–P21) based on processed log_2_ intensity values from 29 biomarkers. The log_2_ intensity values are shown in the Color Key bar scale. The platelet-derived miRNAs are denoted with the prefix ‘pl’ and the micro-vesicular miRNAs are designated as ‘mv’.
(TIF)

Figure S7 **Correlation of platelet-derived miRNA and mRNA targets.** Relationship between the fold change densities of miRNA targets and all transcripts measured from expression profiling of endothelial pinch biopsies from UC vs normal individuals. The p-values denote significant differences in the population of down-regulated genes between miRNA targets and all transcripts by either Bionomial test (*) or by Fisher's Exact Test (**).
(TIF)

Table S1 **Lists of differentially expressed miRNAs identified by SAM from the entire dataset of platelets, PBMC and micro-vesicular fractions at an FDR of 1%.** The “Score” represents the modified t-test statistics calculated by SAM. The “Fold Change” denotes the ratios of the mean intensity in patient samples over control samples. The q-value represents an *estimate* of FDR. This number is typically greater than 0. In our dataset due to the limiting sample size (20 cases and 20 controls) we observe for some features the test statistic to be more extreme than all observed in permutations. Therefore the *estimated* FDR for declaring that feature to be significant is set to 0.
(XLSX)

Table S2 **List of 31 biomarkers derived from the platelet fraction.**
(XLSX)

Table S3 **List of 6 biomarkers derived from the micro-vesicle fractions.**
(XLSX)

Table S4 **List of 29 biomarkers derived from a combination of platelets and micro-vesicular fraction.**
(XLSX)

Table S5 **List of 4 principle miRNA clusters derived from the platelet biomarker panel showing correlated expression profiles and genomic coordinates of the miRNAs.**
(XLS)

Table S6 **List of predicted mRNA targets of the 31 biomarkers identified by TargetScan and DIANA.**
(XLS)

Table S7 **List of enriched GO and Pathway analysis terms and mRNA targets in each significant category.** The percentage of overlap and the overlapping entities of the top groups/pathways ranked by the most significant p-values are shown.
(XLS)
