# Supplementary figures and images for: Genome-Wide Maps of Circulating miRNA Biomarkers for Ulcerative Colitis
Source: PLoS One. 2012 Feb 16;7(2):e31241. doi: 10.1371/journal.pone.0031241 (PMC3281076; doi:10.1371/journal.pone.0031241)

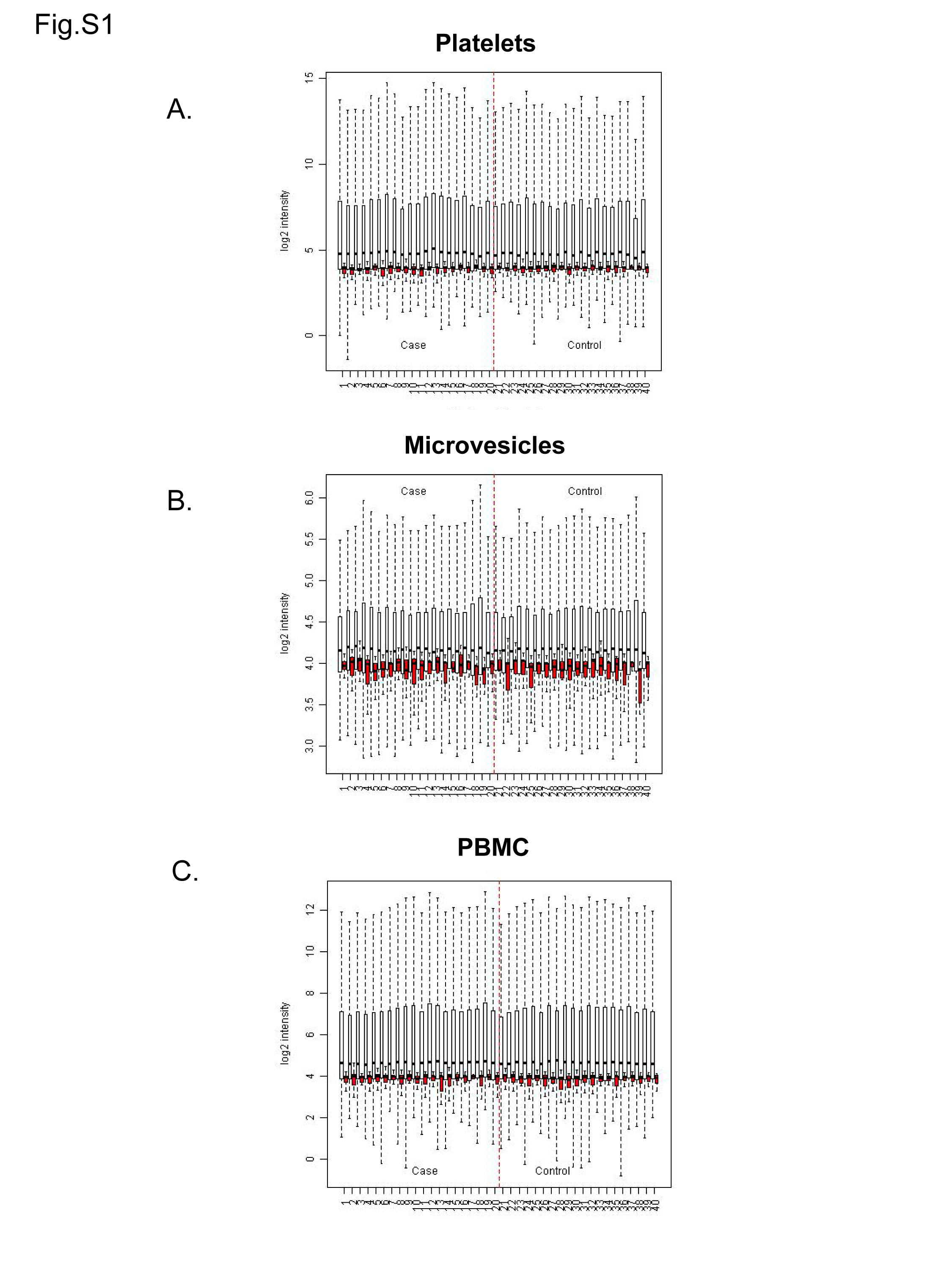

Supplement: Figure S1 — Box plot of log2 signal intensity distribution of human miRNAs (white) and background probes (red) for 20 patients and 20 controls for each fraction (A) Platelets (B) Micro-vesicles (C) PBMC after background subtraction, quantile normalization and median summarization. The black bar represents the median of each distribution and the dashed lines represent the box–plot range set by the whiskers. (TIF) [file pone.0031241.s001.tif]

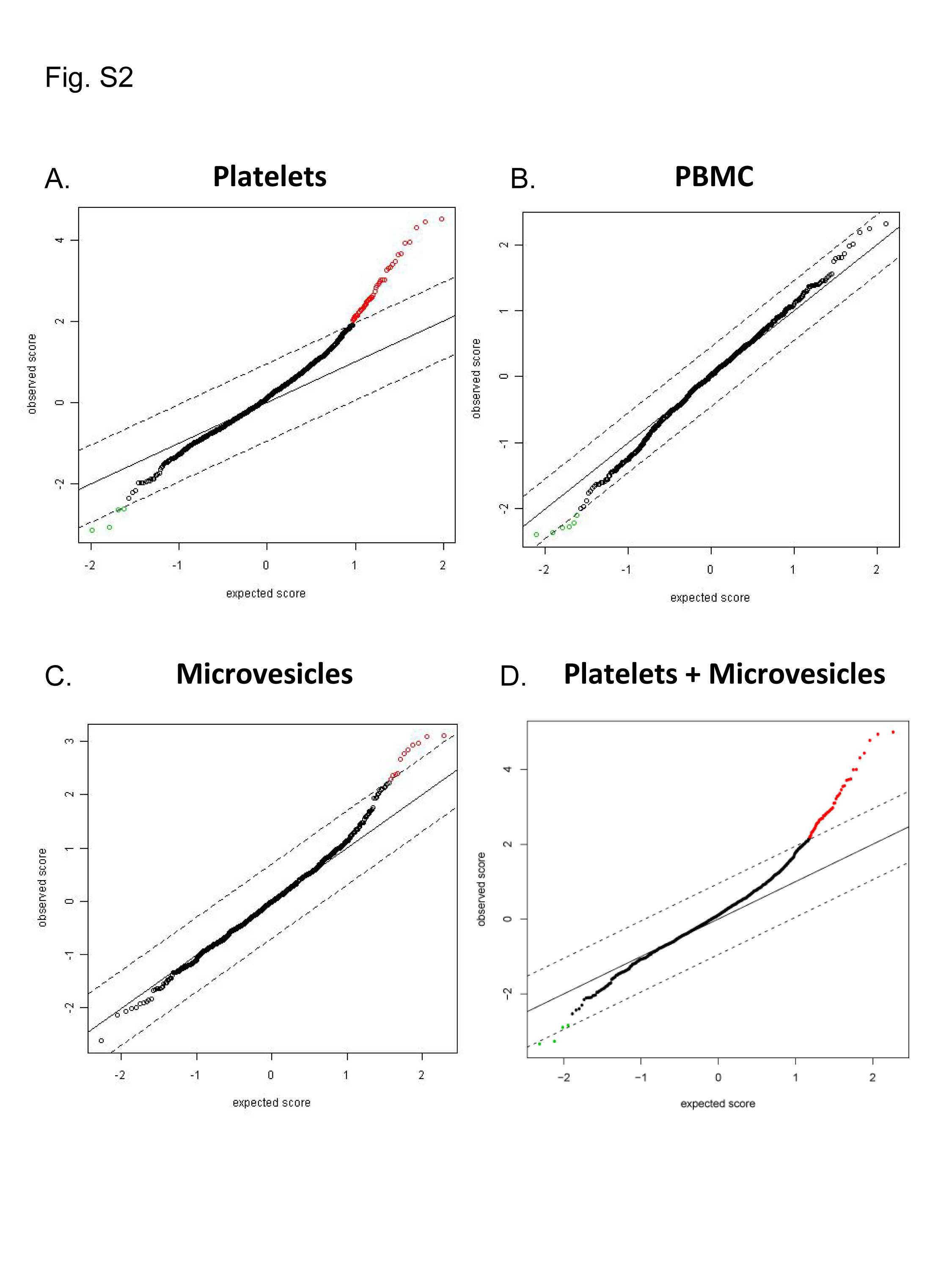

Supplement: Figure S2 — Analysis of differentially expressed miRNA biomarkers from (A) Platelets (B) PBMC (C) Micro-vesicular and (D) Combined Platelet and Micro-vesicle fractions. Comparison of observed vs. the expected scores obtained by performing Significance analysis of microarrays on all 847 miRNAs from 20 patients and 20 controls. Each miRNA is represented by a point (open circle), and the differentially expressed miRNAs represented as red (for up-regulated) or green (for down-regulated) points in the graph. The dashed line represents a FDR threshold of 1%. (TIF) [file pone.0031241.s002.tif]

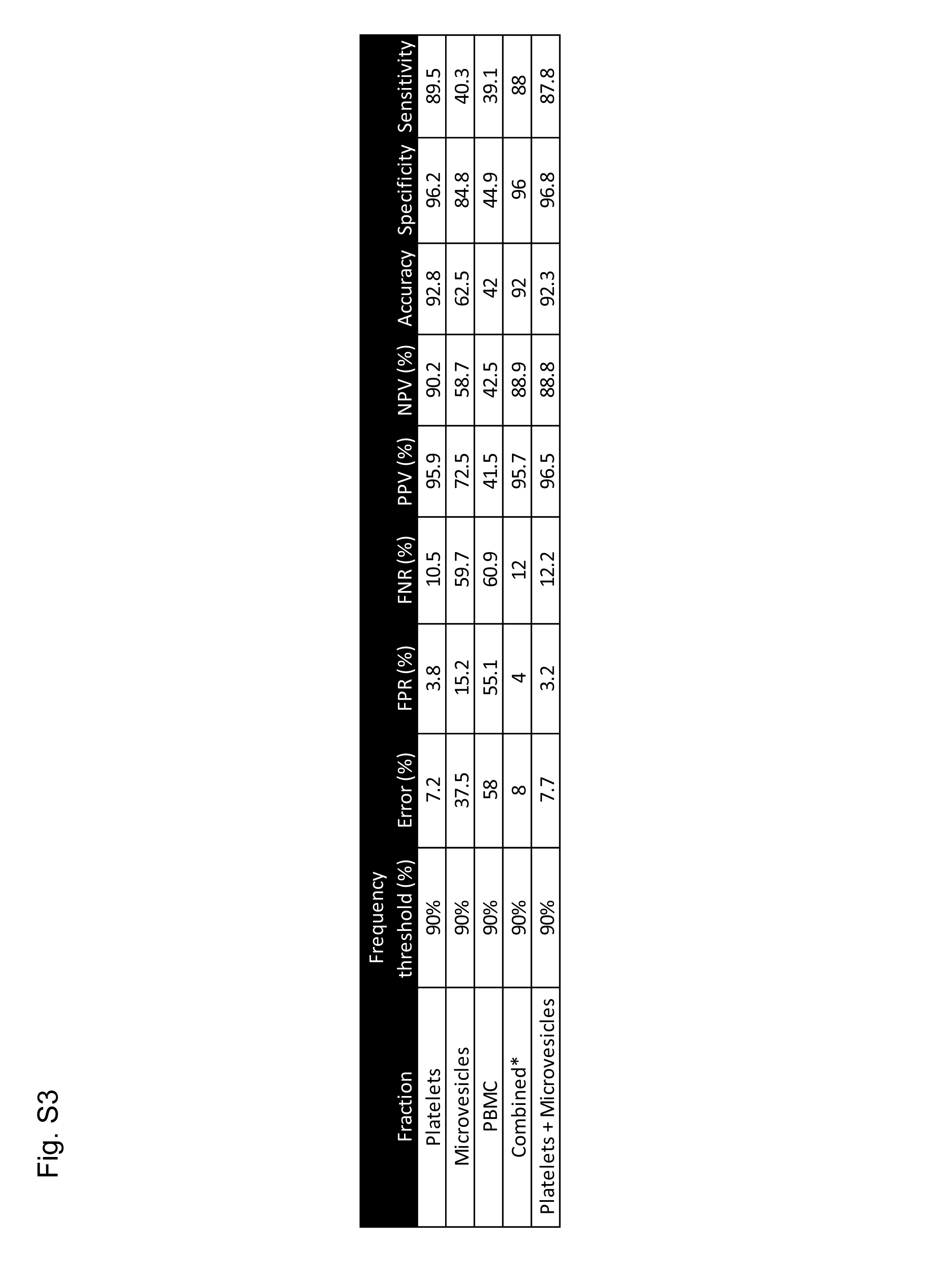

Supplement: Figure S3 — Performance estimates for the different fractions isolated in this study at a frequency cutoff of 90%. The Combined fraction denoted by asterisk is representative of the union of all the 3 different fractions of PBMC, micro-vesicles and platelets. The different error rate calculations are given by FPR (False Positive Rate), FNR (False Negative Rate), PPV (Positive Prediction Value) and NPV (Negative Prediction Value). (TIF) [file pone.0031241.s003.tif]

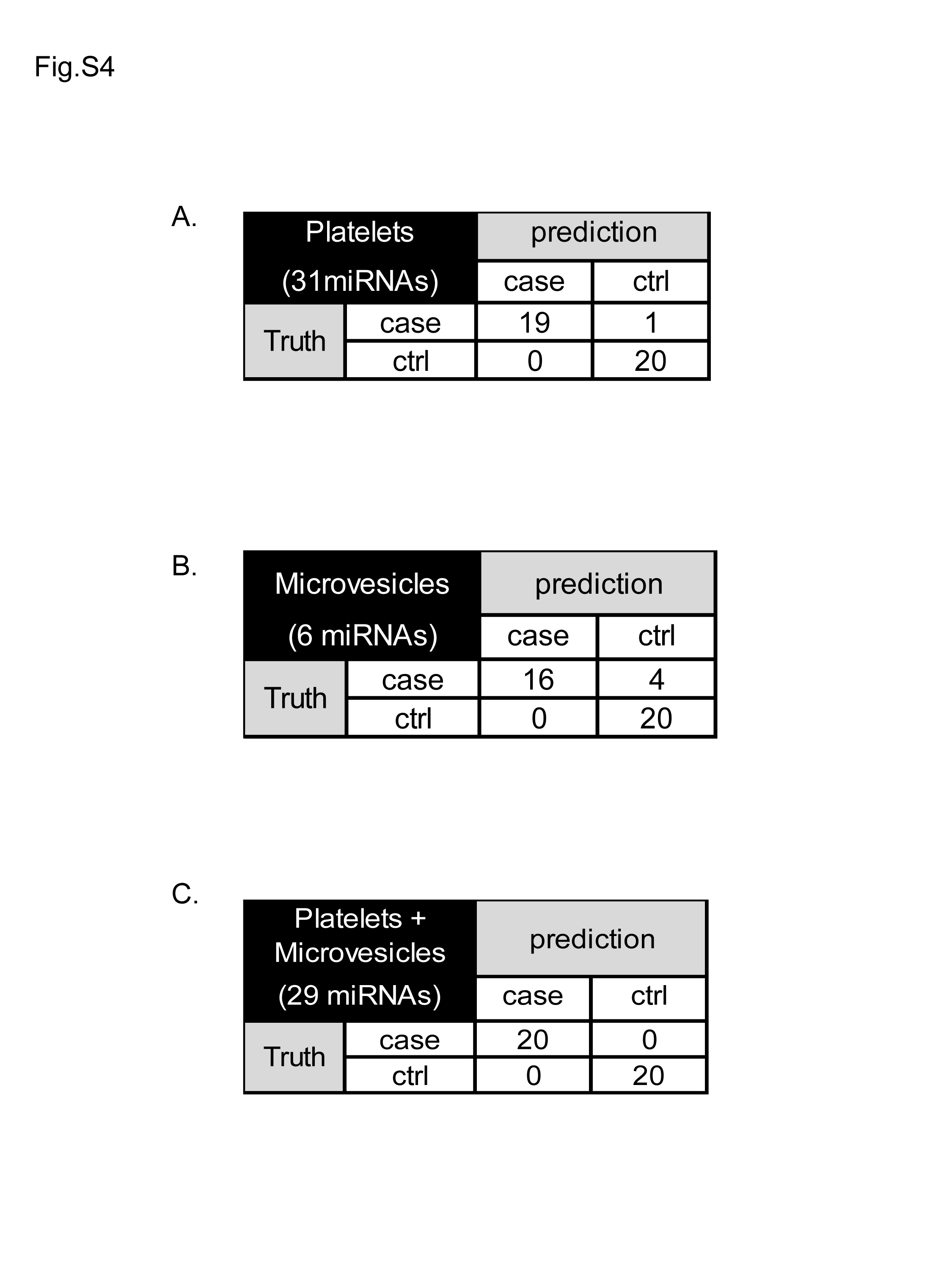

Supplement: Figure S4 — Table of predicted and observed values from each analyzed fraction based on Confusion Matrix derived from SVM on the full data comprising 20 cases and 20 controls. (TIF) [file pone.0031241.s004.tif]

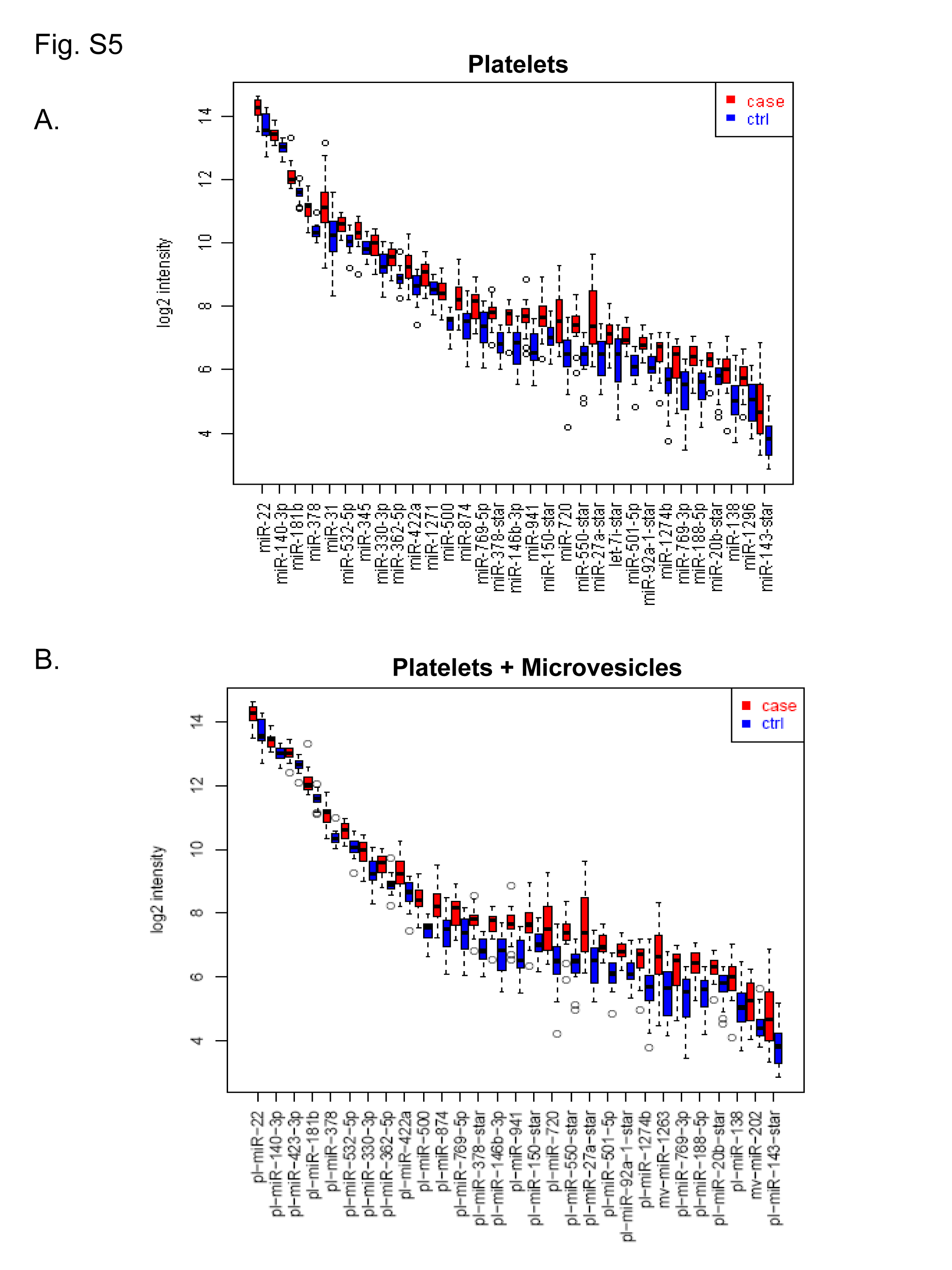

Supplement: Figure S5 — miRNA expression levels in patient and control cohorts. (A) Box plot of processed log2 intensity distributions of 31 miRNA biomarkers from Platelet fraction or (B) 29 miRNAs from the Platelet/Micro-vesicle combined fraction. The black bar represents the median of each distribution and the dashed lines the box-plot ranges. The open circles represent the outliers. The patient samples are denoted by red (cases) and normal samples by blue (controls). The platelet-derived miRNAs are denoted with the prefix ‘pl’ and the micro-vesicular miRNAs are designated as ‘mv’. (TIF) [file pone.0031241.s005.tif]

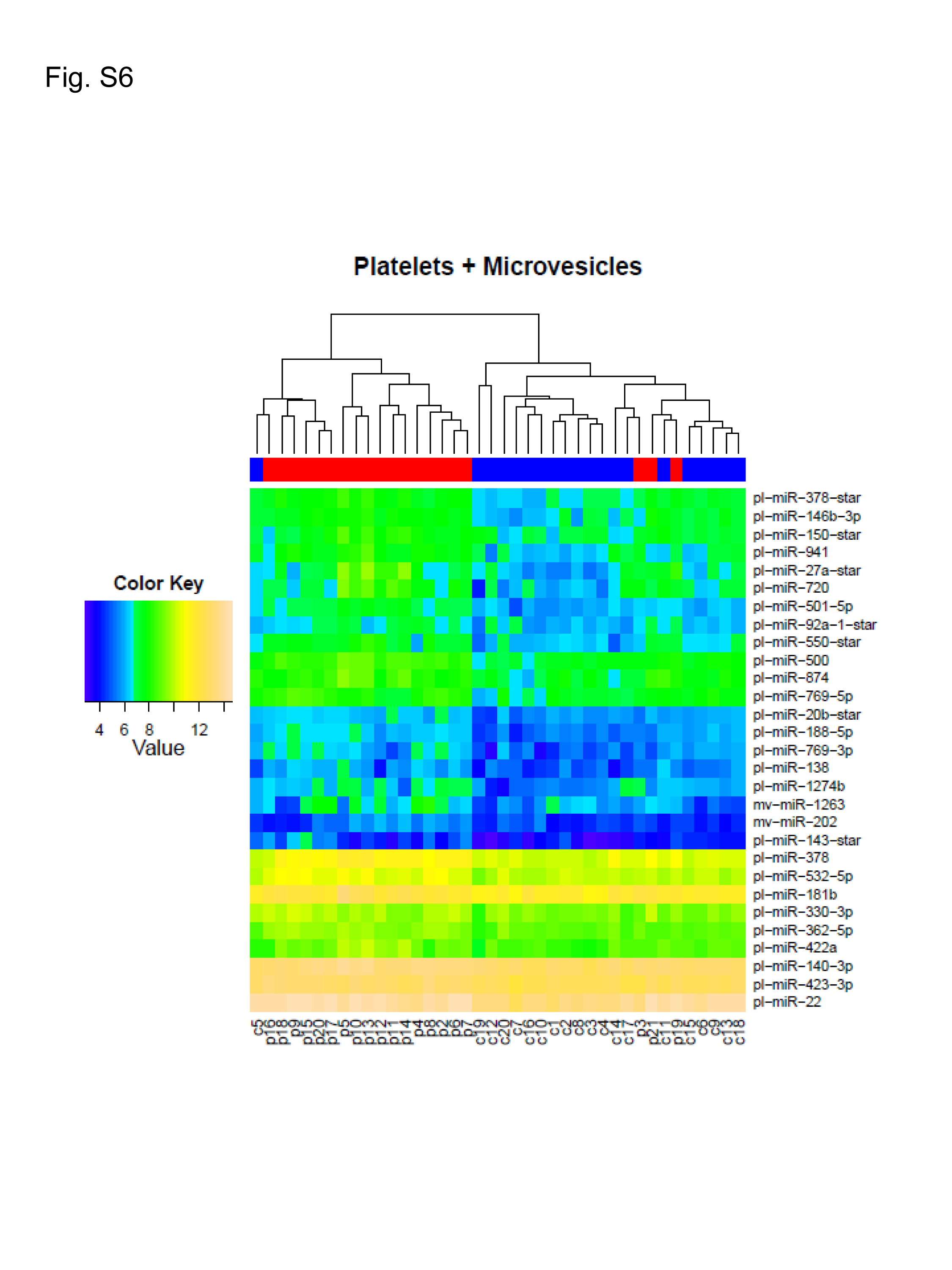

Supplement: Figure S6 — Comparison of expression levels of miRNAs biomarkers in the patient and control cohorts derived from the combination of platelet and microvescicle fractions. Unsupervised hierarchical clustering of samples (controls in blue: C1–C20 and patients in red: P2–P21) based on processed log2 intensity values from 29 biomarkers. The log2 intensity values are shown in the Color Key bar scale. The platelet-derived miRNAs are denoted with the prefix ‘pl’ and the micro-vesicular miRNAs are designated as ‘mv’. (TIF) [file pone.0031241.s006.tif]

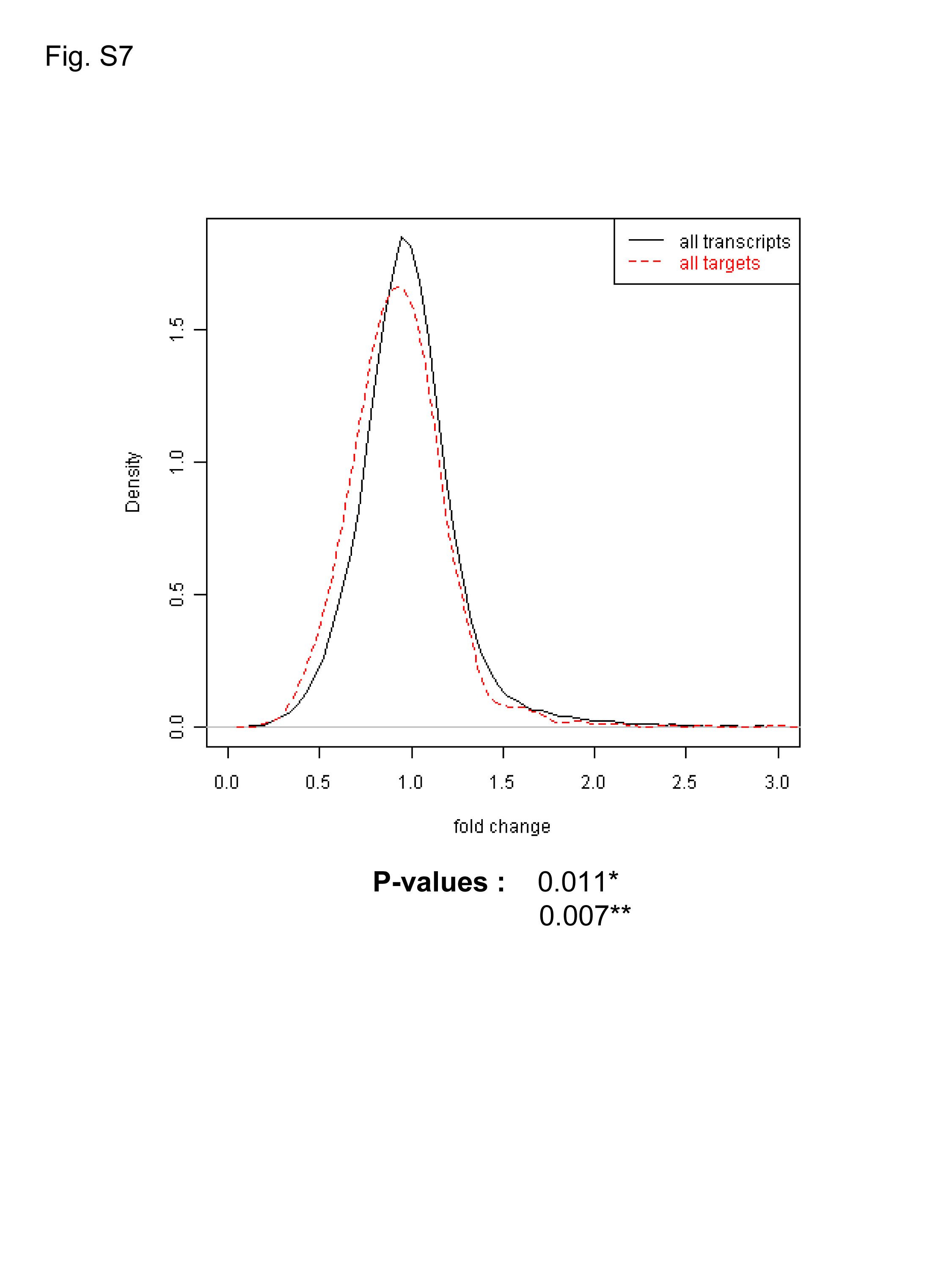

Supplement: Figure S7 — Correlation of platelet-derived miRNA and mRNA targets. Relationship between the fold change densities of miRNA targets and all transcripts measured from expression profiling of endothelial pinch biopsies from UC vs normal individuals. The p-values denote significant differences in the population of down-regulated genes between miRNA targets and all transcripts by either Bionomial test (*) or by Fisher's Exact Test (**). (TIF) [file pone.0031241.s007.tif]
